# How do microtine rodent abundance, snow and landscape parameters influence pine marten *Martes martes* population dynamics?

**DOI:** 10.1002/ece3.70201

**Published:** 2024-08-21

**Authors:** Siow Yan Jennifer Angoh, Petter Johannes Nergaard, Torfinn Jahren, Morten Odden, Scott Michael Brainerd

**Affiliations:** ^1^ Inland Norway University of Applied Sciences Koppang Norway

**Keywords:** agriculture, *Martes martes*, mature spruce forests, microtine rodents, population dynamics

## Abstract

The pine marten (*Martes martes*) occupies the northernmost extent of its distribution in Norway, where microtine rodents are an important food item. The relationship between microtine rodent abundance and pine marten population dynamics is not well understood. In this paper, we examined this relationship and tested if environmental factors (e.g. snow depth, elevation, mature spruce forest density and agricultural land density) modulate pine marten population dynamics. We calculated pine marten abundance indices using data collected from 593 unique snow transects surveyed between 2003 and 2014 in Hedmark, Norway. We employed a Partial Rate Correlation Function to identify potential cyclicity in pine marten populations. We did not observe any cyclical patterns in pine marten populations within our short time series. Instead, their population appeared to be directly density‐dependent. Although the population growth rate of pine marten tended to increase with increasing elevation, it was not affected by individual variables including a microtine rodent abundance index and snow depth. However, the annual growth rate of pine marten populations was positively affected by the interaction between the microtine rodent abundance index and increasing elevation. Pine marten abundance increased with microtine rodent abundance, elevation, snow depth and density of mature spruce forest, but decreased with increasing agricultural land density. Pine martens are opportunistic diet generalists that can switch between prey and cache food for later consumption. They are also strongly territorial with delayed implantation and are slow to respond to environmental changes due to their relatively low reproductive potential. These life‐history traits may mitigate the effects of fluctuating microtine rodent abundance on pine marten reproduction and survival. Nevertheless, our findings suggest that microtine rodents still serve as important prey which can influence the population dynamics of pine martens in higher elevation habitats where alternative prey may be less available.

## Graphical abstract

We found that pine marten populations were directly density dependent in Hedmark, Norway. Additionally, pine marten abundance was positively correlated with a microtine rodent abundance index, elevation, snow depth and mature spruce forests. Their annual population growth rate was positively influenced by the interaction between the microtine rodent abundance index and increasing elevation. These findings emphasize the importance of microtine rodents as a key food source for pine martens, especially in higher elevation areas with less alternative prey.

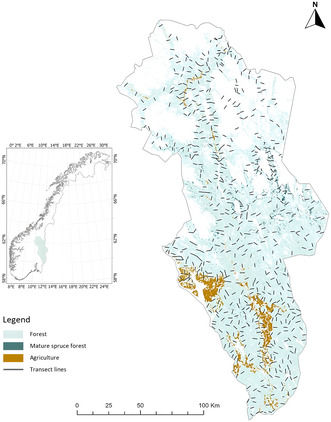

## 1 INTRODUCTION

Among different extrinsic factors, food availability has emerged as a prominent determinant of population dynamics in congeneric *Martes* species (Flynn et al., 2004; Granata et al., 2021; Lensink et al., 1955; Zalewski, 2005). For instance, Flynn and Schumacher (2009) found a strong temporal pattern in annual survival of American martens (*Martes americana*) associated with long‐tailed vole (*Microtus longicaudus*) abundance. In Ontario, population growth rates and densities of American martens changed in correspondence with oscillating densities of small mammals (e.g. snowshoe hare (*Lepus americanus*), southern red‐backed voles (*Clethrionomys gapperi*), deer mice (*Peromy maniculatus*), American red squirrel (*Tamiasciurus hudsonicus*) and flying squirrels (*Glaucomys sabrinus*); Fryxell et al., 1999; Thompson & Colgan, 1987). Some populations of other mesocarnivores such as stoats (*Mustela erminea*), least weasels (*Mustela nivalis*) and red foxes (*Vulpes vulpes*) in northern Europe are synchronized with rodent cycles (Aunapuu & Oksanen, 2003; Korpimäki et al., 1991; Lanszki et al., 2007; Lindström et al., 1995; Storch et al., 1990). In the northern coniferous belt of Sweden, which is characterized by considerable variation in food availability and quality, previous studies observed a strong relationship between the abundance of rodents and the litter sizes and juvenile mortality rates of red foxes (Englund, 1980; Lindström, 1994).

Pine martens (*Martes martes*) are diet generalists that consume small mammals, birds, insects, fruits and carrion (Grupe & Krüger, 1990; Helldin, 1999; Posluszny et al., 2007; Storch et al., 1990; Twining et al., 2019). However, they can display a functional response to the availability of microtine rodents such as bank voles (*Myodes glareolus*), field voles (*Microtus agrestis*) and lemmings (*Lemmus lemmus*) (Helldin, 1999; Jędrzejewski et al., 1993; Rosellini et al., 2008; Zalewski, 2005). This raises questions regarding how pine marten population size and structure might respond to fluctuations in prey abundance. Pine marten population dynamics may be linked to cyclic variations in the populations of main microtine prey species (Helldin, 1999, 2000; Jędrzejewski et al., 1993; Pulliainen & Ollinmaki, 1996). Microtine rodent populations in boreal Scandinavia and Eurasia exhibit cyclic patterns and fluctuate in abundance in 3–5 years intervals (Elton, 1924; Hagen, 1952; Ims & Andreassen, 2000; Kleef & Wijsman, 2015; Korpimäki et al., 1991; Krebs, 2013; Lindström et al., 2001). However, the relationship between the population dynamics of pine martens and microtine rodent fluctuations remains unclear.

In Poland, researchers found a positive correlation between pine marten population density and the abundance of forest rodents the previous autumn (Zalewski, 2007; Zalewski & Jedrzejewski, 2006). However, in south‐central Sweden, Helldin (1999) found that microtine rodent fluctuations did not significantly impact pine marten reproductive success and proportion of yearlings between 1988 and 1997. This lack of demographic response might have been due to the lower amplitudes of microtine rodent cycles across Scandinavia during their study period (Helldin, 1999; Hörnfeldt, 2004). Pine marten populations may not be negatively impacted by periods of low microtine rodent abundance since they can readily switch to alternative sources of nutrition (De Marinis & Masseti, 1995; Jędrzejewski et al., 1993; Pulliainen & Ollinmaki, 1996; Zalewski et al., 1995). For example, when microtine rodent populations are low, pine martens switched to alternative prey species including red squirrels (*Sciurus vulgaris*) (Lindström, 1989; Storch et al., 1990), hares (*Leporidae*), capercaillie grouse (*Tetrao urogallus*) and black grouse (*Lyrurus tetrix*) (De Marinis & Masseti, 1995; Elton, 1924; Hagen, 1952; Hörnfeldt, 1991; Zalewski, 2005). This functional response could act as a stabilizing mechanism for pine marten populations (Ruette et al., 2015). Nevertheless, in Fennoscandia, during periods characterized by microtine rodent cycles with pronounced amplitudes, pine marten populations might exhibit a measurable numerical response over time (Andreassen et al., 2021; Hörnfeldt, 2004). Andreassen et al. (2019) also found that microtine rodent populations fluctuated with greater amplitudes at higher elevations in Norway. This may have a stronger influence on pine marten population dynamics relative to lower elevations.

Snow depth can also modulate accessibility to microtine rodent, with implications for the population dynamics of their predators (Lambin et al., 2000; Storch et al., 1990). Lindström and Hörnfeldt (1994) observed a significant negative correlation between snow depth and the presence of small rodents in the red fox diet. In the northern boreal zone of southeastern Norway, Tengmalm's owls (*Aegolius funereus*) experience similar effects of snow cover on their predation on field voles (Jacobsen & Sonerud, 1993). Storch et al. (1990) speculated that pine martens might not be able to access microtine rodents during periods with deep snow in Sweden. Pulliainen and Ollinmaki (1996) found that incidences of microtine rodents in the diet of pine martens decreased with increasing snow cover. Although pine martens may experience reduced hunting success in deeper snow (Pulliainen & Ollinmaki, 1996; Storch et al., 1990), recent research conducted in Norway revealed a positive association between snow depth and pine marten abundance (Cano‐Martínez et al., 2021) and occupancy (Angoh et al., 2023). Pine martens may prefer forested areas with deeper snow to avoid competition with or predation from red foxes, which may experience lower hunting success compared to pine martens with increasing snow depths (see Willebrand et al., 2017).

Pine marten population dynamics might also be affected by other density‐independent factors, such as the availability of different habitat types, including mature forests and agricultural fields. However, the possible influence of these factors on pine marten population dynamics has received limited attention thus far. In southern boreal Scandinavia, radio‐instrumented pine martens preferred mature, spruce‐dominated forests (Brainerd & Rolstad, 2002). These habitats provide several essential features (e.g. canopy cover, snags and arboreal cavities; Esseen et al., 1997; Kuuluvainen, 2009) which serve as feeding grounds and protective cover from predators. These are key to marten survival and reproduction (Brainerd et al., 1995; Brainerd & Rolstad, 2002; Fisher & Wilkinson, 2005; Thompson, 1994). Kurki et al. (1998) found that pine marten abundance declined as the proportion of agricultural land in the landscape increased. Exposure to predators, such as red foxes and golden eagles (*Aquila chrysaetos*), may be higher in open habitats due to limited vertical escape possibilities and protective cover from predators (Brainerd & Rolstad, 2002; Lindström et al., 1995; Lyly et al., 2015; Sulkava et al., 1999).

Although there is a growing body of literature focusing on identifying and understanding various factors influencing the population dynamics of the genus *Martes* (Proulx & Santos‐Reis, 2012), a notable knowledge gap remains regarding the drivers of spatiotemporal variation in pine marten populations in Scandinavia. We hypothesize that microtine rodent abundance and other extrinsic environmental factors influence pine marten population dynamics. To address our hypothesis, we tested the following predictions: (1) the abundance of microtine rodent, as an important prey species, positively influences both the annual population growth rate and the abundance of pine martens, (2) the responsiveness of pine marten populations to changes in microtine rodent abundance is more pronounced at higher elevations where microtine rodent populations fluctuate with greater amplitudes (3) pine martens benefit from areas with deeper snow where they can evade red foxes, (4) pine marten abundance is related to the density of spruce forests (positive correlation) and agricultural land (negative correlation) due to the adaptation of pine marten to forested habitats and their avoidance of open areas.

## 2 METHODS

### 2.1 Study area

**FIGURE 1:**
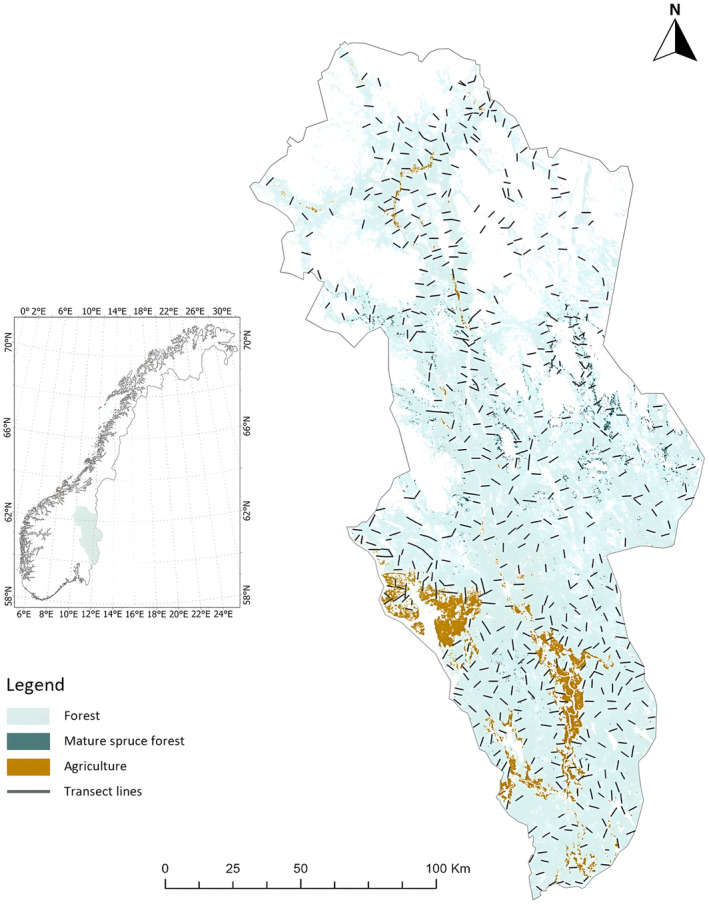
Map of study area within Hedmark showing forest and agricultural land cover. Black lines are locations of snow transects (*n* = 593) on which pine martens were surveyed. Inset of Norway with the Hedmark region in the southeast.

Our study was conducted in the former Hedmark County, Norway, between 2003 and 2014. This region, which has since become part of the larger Innlandet County as of 2020, covers an area of 27,400 km^2^ (Figure 1). Characterized by a clear latitudinal productivity and elevation gradient, the Hedmark area is comprised of highly productive agricultural lowlands mixed with extensive forested regions on low hills in the southern part, transitioning to a less productive landscape in the north. In the northern part, the terrain features deeper valleys, forest ridges and mountains, with agricultural activities and human settlements mostly limited to valley bottoms (Jahren et al., 2020). The forested areas within the study region, totalling 13,000 km^2^, are composed of a mix of deciduous and coniferous species, forming a mosaic of clearcuts, regenerating stands and mature stands (Gustafsson et al., 2010; Jahren et al., 2020; Nybø et al., 2012). Scots pine (*Pinus sylvestris*) and Norway spruce (*Picea abies*) dominate the forests, with deciduous trees such as white birch (*Betula pubescens*) and aspen (*Populus tremula*) scattered throughout (Bendiksen et al., 2008). The elevation varies from 104 to 2119 m above sea level. The climate exhibits semi‐continental characteristics, with the northern zone experiencing harsher conditions compared to the south (Jahren et al., 2020). During the study period (2003–2014), the annual mean temperature ranged from −0.6°C to 3.1°C in Hedmark (CCKP, 2021).

### 2.2 Pine marten census

Between 2003 and 2014, volunteers from the Hedmark Chapter of the Norwegian Association of Hunters and Anglers conducted annual snow transect surveys during the month of January (see Tovmo & Brøseth, 2014; Figure 1). These snow transects were originally devised to monitor family groups of Eurasian lynx (*Lynx lynx*; Linnell et al., 2007). Volunteers monitored three to four snow transects per 100 km^2^. They also recorded tracks of various other mammals, including pine martens (Tovmo & Brøseth, 2014). From a cumulative total of 593 unique snow transects (annual x̄ = 277.83 snow transects ±135.03 SD), volunteers tallied 4205 pine marten crossings. They also recorded corresponding information including snow transect length (x̄ = 2.93 km ± 0.51 SD) and the time elapsed after the most recent snowfall (x̄ = 3.70 days ± 1.65 SD). We used these snow tracking data to obtain a credible approximation of pine marten abundance to infer population dynamics of this species (Kawaguchi et al., 2015; Kurki et al., 1998; Thompson et al., 1989).

We calculated the following annual pine marten abundance index for each snow transect:

(1) $$\text{Pine marten abundance index}=\frac{\text{pine marten tracks}}{\text{transect length}\times \text{days since last snowfall}}$$

### 2.3 Cyclicity diagnosis

We used a diagnostic tool (i.e. partial rate correlation function; PRCF) to detect potential density‐dependent feedback/cyclicity in pine marten populations. The PRCF is an autocorrelation function that regresses the logarithmic per capita rate of change to lagged population densities and provides an estimate of the order of the autoregressive process (Berryman, 1999; Berryman & Turchin, 2001). PRCF analyses require consecutive time series (i.e. observations for each time‐step) to converge. Since not all snow transects were surveyed every year during the study period, we grouped transects (by proximity) into 60 different clusters of approximately 10 transects using the spatstat package (Baddeley et al., 2022) in R version R‐4.3.1 (R Core Team, 2023). Each transect group with >3 time‐steps (*n* = 58) was checked for cyclicity in negative feedback processes using the PRCF. We averaged pine marten abundance indices (see pine marten census section 2.2 for index calculation; Equation 1) by transect groups and used these in the PRCF. We interpreted the residuals from the PRCF model as the amount of change in abundance of a population and values close to zero indicated marginal change (Berryman & Turchin, 2001; see Appendix A). We assessed significance of the correlation coefficients at different lags with Bartlett's criterion test statistics (Bartlett, 1974).

### 2.4 Statistical analyses

#### 2.4.1 Population growth rate model

We used the instantaneous rate of increase (i.e. population growth rate; *R*_*t*_) as a response variable in our model investigating temporal variation in population growth. We calculated the growth rate of the pine marten population by using the formula *R*_*t*_ $=\ln\frac{\mathrm{Nt}}{\mathrm{Nt}-1}$, where N_*t–*1_ represents the pine marten abundance index (see pine marten census section 2.2) lagged by 1 year. The response variable in our model was the average annual pine marten growth rate for each group of snow transects from the year 2008 to 2014. We assessed the relationships of microtine rodent abundance and environmental factors to pine marten population growth rate by fitting a generalized linear mixed model (GLMM) with a normal distribution in a Bayesian framework (see model implementation section 2.6). Several explanatory variables were incorporated into the model, including a microtine rodent abundance index (*rodent. index* covariate), elevation (*elevation* covariate), interaction between the microtine rodent abundance index and elevation (*rodent*×*elevation* covariate) and snow depth (*snow* covariate). To account for potential spatial correlations, we included group ID as a random effect in the model.

#### 2.4.2 Abundance model

To examine the influence of the above explanatory variables (i.e. *rodent. index*, *elevation*, *rodent*×*elevation*, *snow*) on the spatial variation in abundance of pine martens, we fitted a GLMM with negative binomial distribution (see model implementation section 2.6). In addition, mature spruce forest density (*spruce.density* covariate) and agricultural land density (*agri.density* covariate) were added to this model as mature spruce forests and agricultural land have been related to pine marten habitat use in previous studies (Brainerd & Rolstad, 2002; Kurki et al., 1998). The number of pine marten tracks observed per snow transect between 2007 and 2014 was the response variable in this model. To address potential confounding factors related to track length and accumulation over time (Hilbe, 2014), we incorporated log of days since last snowfall and track length as offsets in the model. To address temporal correlations in this model, we added year as a random effect. We also tested for zero‐inflation and evaluated the use of a spatial autocorrelation structure in this model (R script and data available at https://doi.org/10.5281/zenodo.11181197) but did not include these in the final model as they did not improve our analyses.

### 2.5 Covariates

#### 2.5.1 Microtine rodent abundance index

Microtine rodents found in our study area include bank voles, field voles and lemmings. As part of an annual grouse survey (every August from 2006 to 2014), volunteers recorded whether or not they saw live microtine rodents (presence/absence; no species level distinction) along line transects. A peak in forest‐dwelling microtine rodents (i.e. bank vole and field vole) occurred in the autumn of 2011 and 2014 (Breisjøberget et al., 2018). However, as pine marten tracks were not recorded in early 2015, we could not access the effect of the later microtine rodent peak on pine marten dynamics. Therefore, we only used microtine rodent autumn observations from 2006 to 2013.

We obtained line transect data collected across 19 municipalities in Hedmark county (see Appendix B for number of line transects per year and per municipality). We used microtine rodent data from an average of 1557.3 line transects (± 334.9 SD) per year. The average length of each line transect was 3.2 km (± 1.1 SD). We estimated the proportion of line transects within a municipality on which microtine rodent presence was detected and used this as a conservative proxy of microtine rodent abundance.

Since microtine rodent and pine marten data were collected from transects that did not overlap, we spatially interpolated the microtine rodent abundance index throughout Hedmark county using an inverse distance weighting (IDW) tool within ArcGIS Pro version 3.1.2 (ESRI, 2023). We used the microtine rodent abundance index per municipality and respective municipality centroids (with relatively homogeneous spatial distribution across study area; Appendix C) for this interpolation. We selected the default power value of 2 and search neighbourhood (i.e. standard) for creating the interpolated surface. We obtained a raster heatmap, with spatial resolution of 1.9 km (X) by 3.0 km (Y), containing predicted microtine rodent abundance indices for each survey year across the entire study area (Appendix C). Subsequently, we extracted an annual microtine rodent abundance index value (*rodent.index* covariate) from the raster heatmap at the centroid of each pine marten snow transect (see pine marten census section 2.2).

We validated our microtine rodent abundance index by comparing a subset of our rodent data from Engerdal municipality to yearly snap trapping data (Norwegian Environment Agency, 2024) from Gutulia, an area within Engerdal municipality. We used the Hmisc package (Harrell & Dupont, 2024) in R and found that the microtine rodent abundance index in Engerdal municipality correlated with the snap trapping estimated rodent density (calculated as number of catches per 100 trap days) in Gutulia (*R* ^2^ = 0.837, *p* = .019; see Appendix D for values used in the correlation test). Selås et al. (2021) found that in two areas (Varaldskogen and Vangsåsen) within Hedmark county bank vole populations peaked in 2010. We also recorded a peak in our data in 2010 (Appendix C). Moreover, we acknowledge that using an interpolated surface created with the IDW tool may introduce inherent spatial biases due to our use of default parameters. However, in our study area, we found that annual variation in microtine rodent abundance was more pronounced than spatial variation. This may buffer against possible spatial biases in subsequent analyses using indices from our interpolated raster surface.

#### 2.5.2 Environmental variables

While conducting the annual snow transect surveys described above (see pine marten census section 2.2), volunteers recorded snow depth for each transect (*snow* covariate; see Appendix E for range of snow depth). In order to obtain an elevation value for each snow transect (*elevation* covariate), we created a 7.17 km^2^ grid based on the average home range of pine martens (Brainerd, 1997) and then calculated mean elevation per grid cell using a digital elevation model raster with a 50 m pixel spatial resolution (Kartverket, 2016). We overlaid this grid with the pine marten snow transects and sampled a corresponding elevation value for every transect centroid (see Appendix E for range of elevation values).

We also extracted a transect centroid value for the relative density of mature spruce‐dominated forest (≥ 120‐year‐old forest; >50% spruce; *spruce.density* covariate), and relative density of agricultural fields (*agri.density* covariate). We used the SR16 forest resource map (Astrup et al., 2019) and the N250 land use map (Kartverket, 2017) to estimate mature spruce forest density and agricultural field density respectively. The following methods described in Jahren et al. (2020), mature spruce forest patch size (as ɀ value) and geometrical centre were used to predict planar kernel density for this landscape variable across the study area. We followed the same procedure for estimating the agricultural field density but used agricultural field size (as ɀ value) and geometrical centre. We then sampled mature spruce forest and agricultural field kernel density values for the same snow transect centroids as above (see Appendix E for range of densities).

To ensure comparability across variables, we standardized all continuous explanatory variables by applying a z‐transformation, which involved subtracting the mean and dividing by the standard deviation of each variable. In addition, we assessed the Pearson correlation coefficient for pairs of continuous covariates to address potential collinearity concerns. We confirmed that the correlation coefficients between these variables were below 0.6 to minimize the effects of collinearity (Zuur et al., 2010). All the covariates were extracted using ArcGIS Pro version 3.1.2 (ESRI, 2023) and R version R‐4.3.1 (R Core Team, 2023).

### 2.6 Model implementation

We estimated model parameters for both GLMMs described above (see statistical analyses section 2.4) in a Bayesian framework with Markov chain Monte Carlo (MCMC) sampling. These analyses were performed in R version R‐4.3.1 (R Core Team, 2023) using the rstanarm package (Goodrich et al., 2023). We applied weakly informative priors to all parameters. Specifically, a normal distribution with a mean of 0 and a standard deviation of 2 was used for all intercepts and slopes. For each model, we ran 6000 iterations, including 3000 warm‐up iterations, across four chains. Convergence was assessed through the R‐hat statistic (R̂ <1.01; Vehtari et al., 2021) and trace plots (Brooks & Gelman, 1998). To evaluate the model fit, we performed a posterior predictive check using the bayesplot package (Gabry et al., 2019). We reported the slope estimates for all parameters (i.e. mean values and associated 80% credible intervals) and interpreted any non‐overlapping zero 80% credible intervals (CRI) as evidence of a change (McElreath, 2020; Schenker & Gentleman, 2001) in pine marten growth rate or abundance.

## 3 RESULTS

We detected no cyclic pattern in the pine marten population time series that we analysed. Instead, the PRCF indicated direct density‐dependence between 2003 and 2014, with Lag 1 (PRCF[1]) having the lowest negative residuals (see Appendix A) in the majority of the time series (i.e. 56 out of 58 time series). This order of feedback‐delay shows that pine marten populations were directly density‐dependent. That is, the current pine marten population size was dependent on the previous year's population size.

**FIGURE 2:**
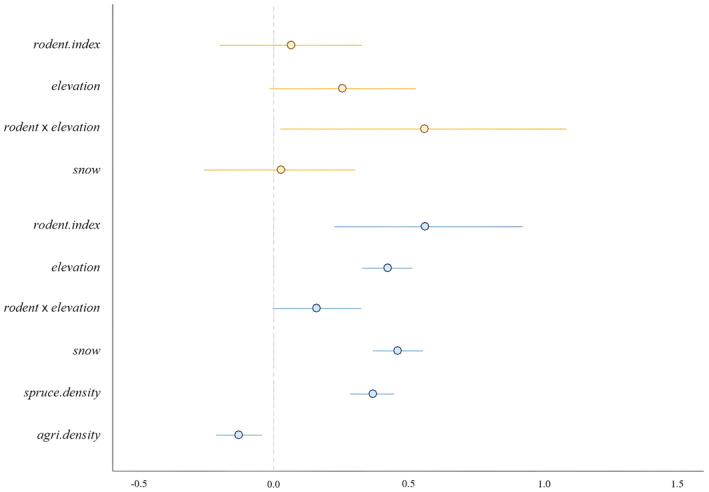
Posterior parameter distributions for models explaining pine marten population growth rate (orange lines) and abundance (blue lines). The horizontal lines represent the 80% credible intervals of the posterior distributions. The circles represent the posterior means.

**FIGURE 3:**
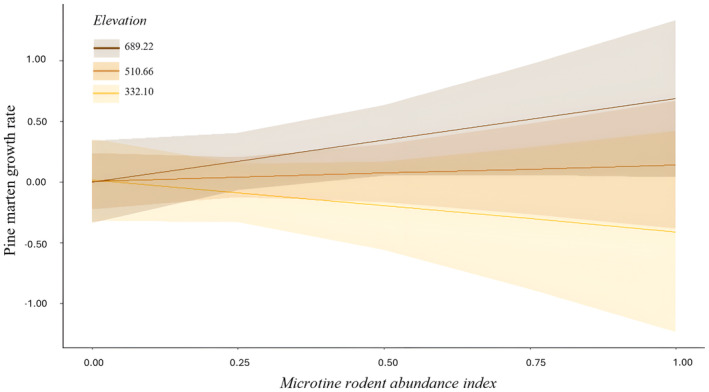
Relationship between pine marten population growth rate and the interaction of the index of microtine rodent abundance with elevation. The standardized elevation values are the mean ± SD (see Appendix E for the range of unstandardized values). The line indicates the mean posterior estimates and includes the 80% credible interval (polygon).

We found no relationship between pine marten population growth rate and the microtine rodent abundance index (β_*rodent.index*_ = 0.065, 80% CRI [−0.200, 0.329]) or snow depth (β_*snow*_ = 0.027, 80% CRI [−0.258, 0.305]; Figure 2). Although CRI overlapped zero, the pine marten population growth rate tended to increase with elevation (β_*elevation*_ = 0.257, 80% CRI [−0.015, 0.532]; Figure 2a). As elevation increased, pine marten population growth rate was positively associated with the index of microtine rodent abundance (β_*rodent*×*elevation*_ = 0.561, 80% CRI [0.026, 1.093]; Figures 2 and 3).

Pine marten abundance was positively correlated with the index of microtine rodent abundance (δ_*rodent.index*_ = 0.566, 80% CRI [0.228, 0.927]), elevation (δ_*elevation*_ = 0.425, 80% CRI [0.330, 0.519]), snow depth (δ_*snow*_ = 0.463, 80% CRI [0.369, 0.558]) and mature spruce forest density (δ_*spruce.density*_ = 0.369, 80% CRI [0.287, 0.449]; Figure 2). At higher elevations, we observed a tendency for pine marten abundance to be positively associated with the index of microtine rodent abundance (δ_*rodent*×*elevation*_ = 0.160, 80% CRI [−0.004, 0.325]; Figure 2). Pine marten abundance and agricultural density were negatively correlated (δ_*agri.density*_ = −0.129, 80% CRI [−0.214, −0.043]; Figure 2).

## 4 DISCUSSION

In this study, we used snow tracking data collected throughout Hedmark county to test for cyclicity in pine marten populations. We also examined how pine marten population growth rate and abundance responded to changes in our index of microtine rodent, elevation, snow depth, mature spruce forest density and agricultural land density. We found no evidence of cyclicity in pine marten populations between 2003 and 2014. Instead, pine marten populations appeared to be directly density‐dependent. Annual growth rates in pine marten populations increased with the microtine rodent abundance index. At higher elevations, pine marten population growth rate and abundance tended to increase with the index of microtine rodent abundance. Pine marten abundance was positively associated with microtine rodent abundance index, as well as elevation, snow depth and mature spruce forest density, while pine marten abundance was negatively correlated with agricultural land density.

First‐order negative feedback was the dominant density‐dependent structure observed in our relatively short time series, indicating that pine marten populations were directly density‐dependent (Berryman & Turchin, 2001) in our study area. A longer time series might have increased the resolution of the data, thereby strengthening the predictive power of our PRCF model in identifying any cyclical population patterns for pine martens. Further investigations using longer time series (e.g. historical pine marten trapping records and corresponding indices of microtine rodent abundance) would be necessary to better understand the relationship between the microtine rodent cyclicity previously recorded within our study area (see Andreassen et al., 2019; Ims & Andreassen, 2000; Selås et al., 2021) and potential corresponding responses in pine marten populations. Nevertheless, our findings corroborate an earlier study that found direct density‐dependence in the congeneric American marten in Ontario, Canada (Fryxell et al., 1999), albeit with little concurrent evidence of rodent cycles in their study area.

The reproductive biology of pine martens, with delayed implantation and late sexual maturation (Helldin & Lindström, 1995; Larroque et al., 2015), may dampen the effects of microtine rodent cycles by regulating population growth (Ferguson et al., 2006) regardless of food availability. Kleef and Wijsman (2015) found that pine marten litter sizes were positively associated with microtine rodent abundance in the Netherlands. However, this variation was low, suggesting that litter size was not strongly affected by fluctuations in microtine abundance. Although there has been little research conducted specifically on how territorial behaviour in pine martens can regulate population size, López‐Sepulcre and Kokko (2005) suggest that territoriality can generally increase population stability. Pine martens in Scandinavia are highly territorial with home ranges overlapping intersexually but not intrasexually (Brainerd, 1997). Through hostile interactions between territory holders, population growth may be limited (Fryxell et al., 1999). Territorial behaviours in pine martens can thus potentially buffer their numerical response regardless of food availability.

Moreover, in Scandinavia, pine martens are considered a diet generalist, and their populations may not track fluctuations in microtine rodent abundance (cf. Helldin, 1999, 2000) as closely as populations of microtine rodent specialists such as stoats and least weasels (Korpimäki et al., 1991). Pine martens prey on microtine rodents when available but can switch over to and supplement their diet with alternate prey, such as red squirrels and birds, during microtine rodent population lows (Helldin, 1999, 2000; Storch et al., 1990; Wijsman, 2012). Helldin (1999) concluded that pine marten population density and demography were independent of microtine rodent cycles. However, the amplitude of cycles, snow depth and a lack of alternative prey could negatively impact pine marten populations, particularly juvenile females that may be more susceptible to the adverse effects of food shortages (Helldin, 1999). During winter, pine martens also retrieve cached food such as eggs, passerine birds, small mammals and amphibians (e.g. Pulliainen & Ollinmaki, 1996; Twining et al., 2018; Willebrand et al., 2017). The caching of food may reduce the reliance of pine martens on microtine rodents and reduce foraging time.

While we did not find a direct relationship between the annual population growth rates of pine martens and the microtine rodent abundance index itself, we did observe a positive correlation with this index with increasing elevation. More pronounced fluctuations in microtine rodent populations have been reported at higher elevations and latitudes (Andreassen et al., 2019; Bjørnstad et al., 1995; Hansson & Henttonen, 1985). Although we did not specifically test for latitudinal effects, within our study area, higher elevation is correlated with latitude (see Jahren et al., 2020). Hence, it is possible that elevation, in conjunction with latitudinal effects contributed to a clearer relationship between pine marten population growth rates and the abundance of microtine rodents. Additionally, animal species diversity generally declines with elevation (Basnet et al., 2016; Rahbek, 1995; Sergio & Pedrini, 2007). A reduced availability of alternative prey at higher elevations might lead to a greater reliance of pine martens on microtine rodents. Moreover, we found that pine marten abundance increased along an elevation gradient. The densities of red foxes, the main enemies and competitors of pine martens (Lindström et al., 1995), tend to be higher in valleys close to human activities (Šálek et al., 2014; Walton et al., 2017). Consequently, pine martens may prefer areas at higher elevations to reduce the risk of competition with red foxes. Indeed Cano‐Martínez et al. (2021) found a negative association between elevation and the abundance of red foxes, while pine marten abundance was positively associated with elevation.

Highlighting a positive relationship between pine marten abundance and snow depth, our results corroborate those of Cano‐Martínez et al. (2021). While martens may be partially limited by increased snow depth, their relatively small body size and light weight‐on‐track may give them an advantage for moving through and over snow (Jędrzejewski et al., 1993; Koehler & Hornocker, 1977; Steventon & Major, 1982). Pine martens can use subnivean spaces to gain access to preferred prey such as *Myodes* voles (Jędrzejewski et al., 1993; Pulliainen & Ollinmaki, 1996). Moreover, deep snow may benefit pine martens as their competitors/predators, red foxes, are disadvantaged in areas with deeper snow (Lindström & Hörnfeldt, 1994; Willebrand et al., 2017).

We also found that pine marten abundance was positively associated with mature spruce forest densities, but negatively associated with densities of agricultural land. This agrees with previous findings reporting that pine martens strongly prefer mature (≥20 m tall) spruce‐dominated forests and avoid open landscapes in southern boreal Scandinavia (Brainerd & Rolstad, 2002; Kurki et al., 1998; Storch et al., 1990). Pine martens may use residual forest patches, hedgerows and riparian hedgerows to disperse (Pereboom et al., 2008). Nevertheless, pine martens prefer areas with access to cover and are rarely found far from forest habitats (Pereboom et al., 2008). Areas with a high density of agricultural land may represent sink habitats (Pulliam, 1988) for pine martens.

## 5 CONCLUSIONS

Our findings indicate that pine marten populations in Hedmark county displayed direct density dependence rather than the delayed density dependence that is typical for cyclic populations (Row et al., 2014), even if our data comprise a relatively short time series. Although pine martens may display a functional dietary response to microtine rodents when they are abundant (Helldin, 2000), numerical response to microtine rodent fluctuations may be dampened due to the pine martens' generalist diet, strong territoriality, caching behaviour and low intrinsic rate of population increase. Moreover, our index of microtine rodent abundance was positively associated with pine marten abundance and pine marten population growth rates increased with microtine abundance as a function of increasing elevation. This suggests that at higher elevations where microtine rodent cycles are more pronounced (Andreassen et al., 2019) and alternative prey may be limited (Sergio & Pedrini, 2007), microtine rodents may have a greater influence on the pine marten population dynamics. However, future research should focus on the functional and numerical responses of pine marten populations to microtine rodent abundance over several consecutive microtine rodent cycles in order to clarify these relationships in boreal Scandinavia.

## AUTHOR CONTRIBUTIONS

**Siow Yan Jennifer Angoh:** Conceptualization (equal); formal analysis (lead); investigation (lead); methodology (lead); visualization (lead); writing – original draft (equal); writing – review and editing (lead). **Petter Johannes Nergaard:** Conceptualization (equal); formal analysis (supporting); methodology (equal); visualization (supporting); writing – original draft (equal); writing – review and editing (supporting). **Torfinn Jahren:** Conceptualization (equal); formal analysis (supporting); investigation (supporting); methodology (equal); supervision (equal); writing – review and editing (equal). **Morten Odden:** Conceptualization (equal); data curation (lead); formal analysis (supporting); funding acquisition (equal); methodology (equal); project administration (supporting); supervision (equal); writing – review and editing (supporting). **Scott Michael Brainerd:** Conceptualization (equal); formal analysis (supporting); funding acquisition (equal); investigation (supporting); methodology (equal); project administration (lead); supervision (equal); writing – review and editing (equal).

## CONFLICT OF INTEREST STATEMENT

The authors declare no competing interests.

## Data Availability

Analyses reported in this article can be reproduced using data and code from Zenodo Digital Repository: https://doi.org/10.5281/zenodo.11181197

## APPENDIX A

### A.1

Example of partial rate correlation function (PRCF) for one of the pine marten population time series.

## APPENDIX B

### B.1

Number of line transects used to detect microtine rodents per year and per municipality.

**Table:**

Municipality	2006	2007	2008	2009	2010	2011	2012	2013
Alvdal	*NA*	*NA*	*NA*	*NA*	13	*NA*	*NA*	*NA*
Eidskog	*NA*	*NA*	*NA*	95	92	49	37	31
Elverum	40	61	56	*NA*	65	*NA*	*NA*	*NA*
Engerdal	NA	116	39	139	146	174	96	131
Folldal	136	172	161	66	105	130	18	22
Grue	*NA*	*NA*	*NA*	*NA*	*NA*	*NA*	22	23
Harmar	97	32	150	126	159	151	109	73
Kongsvinger	52	59	55	37	45	47	*NA*	*NA*
Løten	40	59	60	49	46	47	37	*NA*
Nord‐Odal	*NA*	*NA*	*NA*	*NA*	*NA*	*NA*	*NA*	26
Os	*NA*	57	90	138	86	122	102	*NA*
Rendalen	159	88	67	68	58	182	153	163
Ringsaker	36	53	72	43	35	31	NA	35
Stange	70	85	100	37	57	70	51	56
Stor‐Elvdal	191	149	90	180	228	313	190	182
Tolga	119	37	90	121	128	168	184	191
Trysil	164	*NA*	99	272	307	331	7	*NA*
Tynset	195	367	275	238	237	303	178	303
Åmot	55	76	86	82	65	61	25	16

## APPENDIX C

### C.1

Inverse distance weighting heatmaps (2006–2013) with prediction of microtine rodent abundance index values across Hedmark. Red crosses mark the centroid of each municipality centroid.

## APPENDIX D

### D.1

Microtine rodent indices in Engerdal municipality from 2007 to 2013 and estimated small rodent density values recorded in Gutulia area during the same period.

**Table:**

Year	Engerdal	Gutulia (catches per 100 trap days)
2007	0.026	0.884
2008	0.077	0.000
2009	0.000	0.000
2010	0.116	10.422
2011	0.747	14.774
2012	0.021	0.000
2013	0.008	3.301

## APPENDIX E

### E.1

Descriptive statistics for the environmental covariates used in the models. All covariates were standardized (mean = 0 and st.dev. = 1) for the statistical analyses.

**Table:**

Covariate	Mean (± St.Dev)	Range
*Snow* (cm)	44.939 (± 22.164)	2.000–155.000
*Elevation* (m)	519.578 (± 210.649)	149.672–1071.778
*Spruce. density* (index)	34.046 (± 12.763)	3.797–87.406
*Agri.density* (index)	0.559 (± 0.458)	0.000–2.052
